# Chronic kidney failure mineral bone disorder leads to a permanent loss of hematopoietic stem cells through dysfunction of the stem cell niche

**DOI:** 10.1038/s41598-018-33979-7

**Published:** 2018-10-18

**Authors:** Marina A. Aleksinskaya, Matthieu Monge, Michiel Siebelt, Edith M. Slot, Karin M. Koekkoek, Ruben G. de Bruin, Ziad A. Massy, Harrie Weinans, Ton J. Rabelink, Willem E. Fibbe, Anton Jan van Zonneveld, Melissa van Pel

**Affiliations:** 1 Department of Internal Medicine (Nephrology) and the Einthoven Laboratory for Experimental Vascular Medicine, Leiden University Medical Center, Leiden, The Netherlands; 2 Orthopaedic Research Laboratory, Erasmus Medical Center, Rotterdam, The Netherlands; 3 Department of Immunohematology and Blood transfusion, Leiden University Medical Center, Leiden, The Netherlands; 4 Division of Nephrology, Ambroise Paré Hospital, University of Versailles-Saint-Quentin-en-Yvelines, Boulogne-Billancourt/Paris, and INSERM U-1018, CESP Team 5 (EpReC, Renal & Cardiovascular Epidemiology), Villejuif, France; 5 Department of Orthopaedics, University Medical Center Utrecht, Utrecht, The Netherlands; 6 Department of Biomechanical Engineering, TUDelft, The Netherlands

**Keywords:** Hematopoietic Stem Cells (HSC), Chronic Renal Failure, Hematopoietic Progenitor Cells (HPC), Mineral Bone Disorder (MBD), Cinacalcet, Haematopoietic stem cells, Molecular medicine

## Abstract

In chronic kidney disease (CKD), endothelial injury, is associated with disease progression and an increased risk for cardiovascular complications. Circulating cells with vascular reparative functions are hematopoietic and also reduced in CKD. To explore the mechanistic basis behind these observations, we have investigated hematopoietic stem cell (HSC) homeostasis in a mouse model for non-progressive CKD-mineral and bone disorder with experimentally induced chronic renal failure (CRF). In mice subjected to 12 weeks of CRF, bone marrow HSC frequencies were decreased and transplantation of bone marrow cells from CRF donors showed a decrease in long-term HSC repopulation compared to controls. This loss was directly associated with a CRF-induced defect in the HSC niche affecting the cell cycle status of HSC and could not be restored by the PTH-reducing agent cinacalcet. In CRF, frequencies of quiescent (G0) HSC were decreased coinciding with an increase in hematopoietic progenitor cells (HPC) in the S-and G2-phases of cell cycle. Moreover, in CRF mice, HSC-niche supporting macrophages were decreased compared to controls concomitant to impaired B lymphopoiesis. Our data point to a permanent loss of HSC and may provide insight into the root cause of the loss of homeostatic potential in CKD.

## Introduction

Chronic kidney disease (CKD) is a pathophysiological condition characterized by a progressive loss of kidney function. In CKD, phosphate retention, decreased (active) vitamin D and increased fibroblast growth factor 23 concentrations are the main driving factors that lead to secondary hyperparathyroidism^1^. This dysregulation of the parathyroid gland is characterized by the sustained release of parathyroid hormone (PTH) that drives bone remodeling by increasing osteoclast and osteoblast activities and bone turnover. Particularly in patients with end-stage renal disease, humoral and biochemical disorders lead to the development of CKD-mineral and bone disorder (CKD-MBD) characterized by progressive bone fragility and vascular calcifications^2^.

A hallmark of CKD is endothelial injury, which is associated with both disease progression and an increased risk for cardiovascular disease^3^. The bone marrow (BM) is a source of both hematopoietic stem cells (HSC) as well as endothelial progenitor cells (EPC). Despite controversies regarding their mechanisms of action, evidence is accumulating that multiple BM populations including CD34^+^ precursor cells and myeloid pro-angiogenic cells can promote endothelial repair^4–6^. Circulating numbers of CD34^+^ progenitor cells are markedly reduced in patients with CKD and this decrease directly correlates with decline of kidney function and the progression of cardiovascular complications^7^. The reasons for the loss of circulating BM-derived vascular progenitor cells in CKD patients are poorly understood, but may be related to impairment of the hematopoietic compartment.

Hematopoietic stem cells (HSC) are the only cells that have the enduring capacity to produce all blood cell lineages. They possess self-renewal capacity and reside in specialized microenvironments in the BM. These niches provide tightly controlled signals to maintain HSC properties including quiescence, long-term self-renewal capacity and multipotency^8,9^. HSC proliferation needs to adapt to differential circumstances including steady state hematopoiesis, stress-induced self-renewing proliferation, inflammation and blood loss. These processes need to be tightly regulated as uncontrolled HSC proliferation may lead to stem cell exhaustion^10,11^.

HSC are enriched in the perivascular area of the endosteal region of the BM, in the proximity of Leptin Receptor^+^ or NG2^+^ pericytes, CXCL12-abundant reticular (CAR) cells, Nestin^+^ stromal cells, endothelial cells and immature osteolineage cells^12–19^. Also, at the HSC-enriched endosteal surface, the concentration of calcium ions is increased. HSC express the calcium-sensing receptor (CaSR) and respond to extracellular calcium concentrations. As such, HSC that lack the CaSR migrate out of the bone marrow toward the peripheral blood and spleen and have lost the capacity to engraft in the bone marrow upon transplantation. This suggests that the CaSR plays a role in HSC localization^20,21^. In addition, CaSR-signalling increases CXCR4 signalling in HSC and increases HSC-binding to the extracellular matrix in the hematopoietic stem cell niche.

The pathophysiological mechanisms that underlie the development of mineral bone disease in kidney disease may impact directly on the integrity of the HSC niche. In the BM, the PTH receptor is expressed in cells of the osteoblastic lineage, including Nestin^+^ stromal cells, osteocytes and osteoblasts^14,22^. Osteoblastic cells greatly influence HSC homeostasis. Targeted deletion of osteoblasts resulted in a subsequent loss of HSC, while increased activity of osteoblasts resulted in increased HSC numbers^22–24^. Activation of osteoblastic cells in response to PTH increases several osteoblastic signals including CXCL12 and IL-6 and increases the number of HPC with limited self-renewal capacity in a T cell-dependent manner^22,25^. However, thus far no effect on HSC in CKD-MBD was observed.

Given the endothelial injury and the disturbed osteoblast metabolism in CKD, we have investigated HSC homeostasis in a mouse model for chronic renal failure (CRF). We demonstrate that chronic renal failure (CRF) leads to a significant decrease in HSC repopulating capacity as a result of a functional defect in the HSC niche. This may provide insight into the root cause of the loss of homeostatic potential in CKD.

## Results

### Induction of non-progressive, stable chronic renal failure with features of mineral bone disorder

To confirm the development of CRF in our model, peripheral blood (PB) plasma was analyzed at 6 and 12 weeks following nephrectomy and compared to age-matched, sham-operated controls. All CRF mice showed features typical of CKD, consistent with earlier studies performed with this model (Table 1)^26^. Moreover, from week 6 to 12 after CRF-induction, Lin^−^Sca-1^+^Flk^+^ EPC were significantly decreased in the PB. In contrast, no differences were observed in the Lin^−^Sca-1^+^Flk^+^ population in the BM at week 12 post CRF-induction (Table 1). As a result of CRF, bone structural changes were observed in the metaphysis region, including increased trabecular bone volume density (BV/TV) and decreased trabecular structure model index (SMI) by a tendency to a more plate-like shape (Table 1, Supplementary Fig. 1)^27^. Administration of Cinacalcet from week 6 after CKD induction onwards, reduced PTH levels in the PB with 54% compared to CRF mice that did not receive Cinacalcet (Supplementary Fig. 2A). Moreover, after Cinacalcet administration, the trabecular bone volume and the BV/TV ratio were similar between CKD mice and sham controls, while no effect on the SMI was observed (Supplementary Fig. 2B–D). Thus, CRF mice show features comparable to symptoms of patients in stage III-IV of CKD accompanied by features of mineral bone disorder (MBD). This effect is partially reversed by administration of Cinacalcet.

**Table 1:** Characteristics of peripheral blood- and bone parameters in non-progressive, stable chronic renal failure with features of CKD-MBD.

Characteristics	Baseline	6 weeks			12 weeks		
		Control	CRF	P value	Control	CRF	P value
Urea (mmol/L)	9.83 ± 1.85	12.11 ± 2.22	32.94 ± 5.70	<*0*.*01*	11.27 ± 3.06	29.87 ± 7.27	<*0*.*01*
Creatinine(μmol/L)	15.13 ± 4.00	16.32 ± 2.83	27.64 ± 5.54	<*0*.*01*	19.27 ± 9.49	25.00 ± 11.52	*0*.*02*
Calcium (mmol/L)	2.54 ± 0.18	2.51 ± 0.15	2.82 ± 0.19	<*0*.*01*	2.42 ± 0.22	2.72 ± 0.28	<*0*.*01*
PTH (pg/mL)	ND	ND	ND	ND	140.5 ± 68.7	320.8 ± 202.9	<*0*.*01*
HgB (mmol/L)	9.25 ± 0.74	9.28 ± 1.00	7.77 ± 1.09	<*0*.*01*	9.54 ± 0.71	8.40 ± 1.03	<*0*.*01*
RBC (x10^12^/L)	10.51 ± 0.54	10.11 ± 1.14	9.69 ± 1.06	0.12	10.61 ± 1.14	10.36 ± 1.00	0.44
Trabecular BV/TV (%)	15.02 ± 2.48	13.38 ± 1.50	17.73 ± 0.81	*0*.*02*	11.10 ± 2.16	14.25 ± 2.81	<*0*.*01*
SMI	1.83 ± 0.21	1.93 ± 0.08	1.81 ± 0.07	0.06	2.02 ± 0.12	1.89 ± 0.13	*0*.*02*
Phosphate (mmol/L)	1.86 ± 0.37	1.50 ± 0.30	1.49 ± 0.30	0.59	1.70 ± 0.53	1.64 ± 0.41	0.80
WBC (x10^9^/L)	9.18 ± 2.28	9.95 ± 2.21	10.65 ± 2.65	0.12	9.15 ± 2.85	10.00 ± 3.71	0.25
Granulocytes (%)	4.42 ± 1.26	7.65 ± 1.96	7.18 ± 1.71	0.46	7.95 ± 1.66	7.33 ± 2.26	0.29
B cells (%)	49.33 ± 3.50	54.98 ± 2.88	55.47 ± 3.25	0.81	54.15 ± 3.16	53.55 ± 2.99	0.59
Monocytes (%)	1.43 ± 0.30	2.64 ± 0.87	2.45 ± 0.93	0.40	2.58 ± 0.89	1.95 ± 0.63	*0*.*01*
Macrophages (%)	3.92 ± 0.84	3.42 ± 0.64	2.70 ± 0.59	<*0*.*01*	3.58 ± 0.74	3.10 ± 0.58	*0*.*03*
CD3^+^CD4^+^ T cells (%)	19.85 ± 2.09	10.05 ± 2.04	11.26 ± 1.53	*0*.*03*	11.68 ± 2.51	12.92 ± 2.07	0.11
CD3^+^CD8^+^ T cells (%)	13.67 ± 1.39	10.08 ± 1.07	11.26 ± 1.53	<*0*.*01*	10.90 ± 1.46	11.16 ± 1.39	0.66
PB Sca + Flk + EPC (%)^*^	0.34 ± 0.13	0.48 ± 0.12	0.43 ± 0.10	0.75	0.44 ± 0.26	0.26 ± 0.05	<*0*.*05*
BM Sca + Flk + EPC (%)^*^	ND	ND	ND	ND	0.29 ± 0.22	0.30 ± 0.12	0.69

^*^Percentage in Lineage-negative population.

### Increased CFU-C in peripheral blood of CRF mice

**Figure 1:**
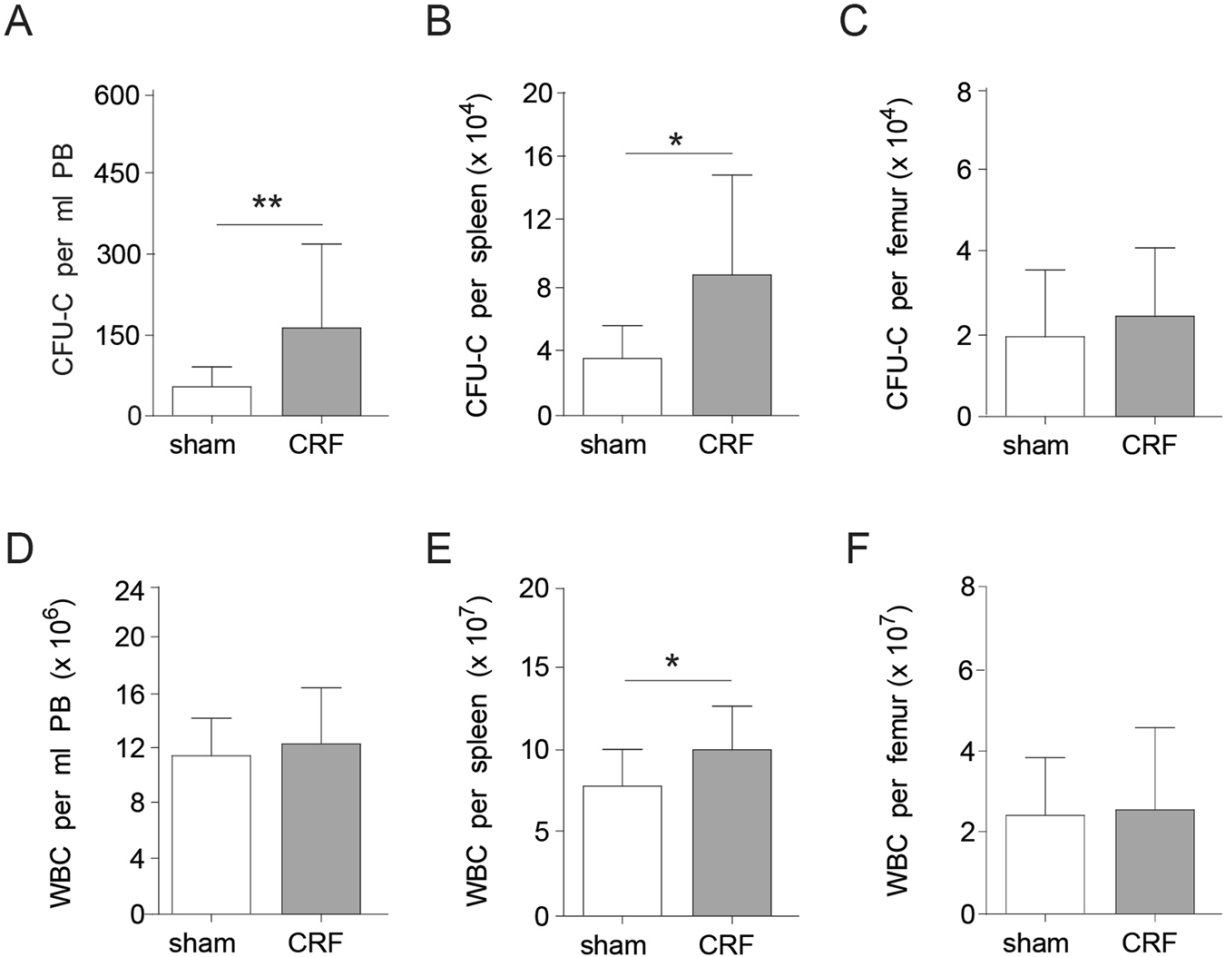
CFU-C levels are increased in the PB of CRF mice. CFU-C were enumerated in the (**A**) PB, (**B**) spleen and (**C**) BM of CRF mice and sham-operated controls (n = 12–16 per group). White blood cell counts per (**D**) ml of PB, (**E**) total spleen and (**F**) femur. Means ± SD are depicted. ^*^p < 0.05 and ^**^<0.01.

To investigate whether the bone-structural changes in our CRF mice affected hematopoiesis, PB was analyzed for the presence of mature blood cell lineages and immature colony-forming units (CFU-C, Fig. 1A). CRF mice had white blood cell numbers at levels similar to controls (Fig. 1D) and no difference was observed in granulocyte and B-lymphocyte frequencies. However, a small, but significant, decrease in monocytes and macrophages was observed, as well as an increase in T-lymphocytes (Table 1). In the bone-lining cells of CRF mice, RANKL is significantly upregulated and M-CSF expression is non-significantly increased. Furthermore, Cathepsin K is upregulated in the BM of CRF mice (Supplementary Fig. 2). In the PB, CFU-C activity was 3-fold increased in CRF, compared to controls (Fig. 1A). A similar increase was observed in the spleen (Fig. 1B), whereas CFU-C in the BM remained at levels similar to controls (Fig. 1C). While white blood cell counts per femur were similar, an increase in splenocytes was observed in CRF (Fig. 1E,F). Increased frequencies of CFU-C in the PB and subsequent migration towards the spleen may point towards pathophysiologic alterations in HSC and their microenvironment^28^. Therefore, these data point towards a possible defect in the hematopoietic compartment of CRF mice.

### Hematopoietic stem cell frequencies are decreased in CRF mice

**Figure 2:**
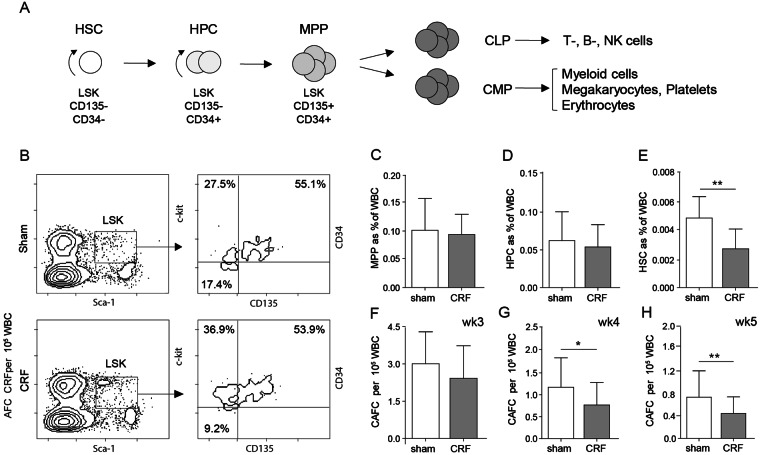
HSC numbers are decreased in the BM of CRF mice. (**A**) Scheme of hematopoiesis and the phenotypes of HSC, HPC and MPP. LSK indicates Lin^−^Sca-1^POS^c-Kit^HI^ cells. Common myeloid progenitor (CMP), common lymphoid progenitor (CLP) (**B**) FACS plots for the identification of stem cell subsets. Representative plots for sham-operated controls (sham) and CRF mice are shown. Using FACS analysis the frequency of (**C**) MPP, (**D**) short-term repopulating HPC and (**E**) long-term repopulating HSC were determined phenotypically (n = 9–13/group). The percentages in the dotplot (**B**) refer to the percentages of MPP, HPC and HSC in the LSK population, while the percentages in the graphs (**C–E**) refer to the percentages of cells with all bone marrow cells. Using a CAFC assay, the frequency of CAFC (**F**) week 3, (**G**) week 4 and (**H**) week 5 was assessed functionally (n = 8 per group). Means ± SD are depicted. ^*^p < 0.05 and ^**^<0.01.

Next we assessed the impact of CRF on the frequency of HSC and HPC using flow-cytometry (Fig. 2A,B) and cobblestone area forming cell (CAFC) analysis. CRF and control mice had similar frequencies of multi-potent progenitors cells (MPP, Fig. 2C) and short-term repopulating HPC (Fig. 2D). In contrast, HSC frequencies were significantly decreased in CRF BM, compared to controls (Fig. 2E). Similar numbers were obtained by the functional enumeration using CAFC analysis. CAFC week 1–3, representing MPP and HPC were at similar frequencies for CRF and controls (Fig. 2F). In contrast, CAFC week 4 and 5 frequencies, representing primitive HSC with long-term repopulating capacity, were significantly decreased in CRF (Fig. 2G,H). Following Cinacalcet administration no differences were observed in the number of LSK, HSC, HPC and MPP in CRF mice (Supplementary Fig. 2E–H).This suggests that other factors than PTH are responsible for the decrease in HSC.

### Long-term repopulating HSC are decreased in bone marrow of CRF mice

**Figure 3:**
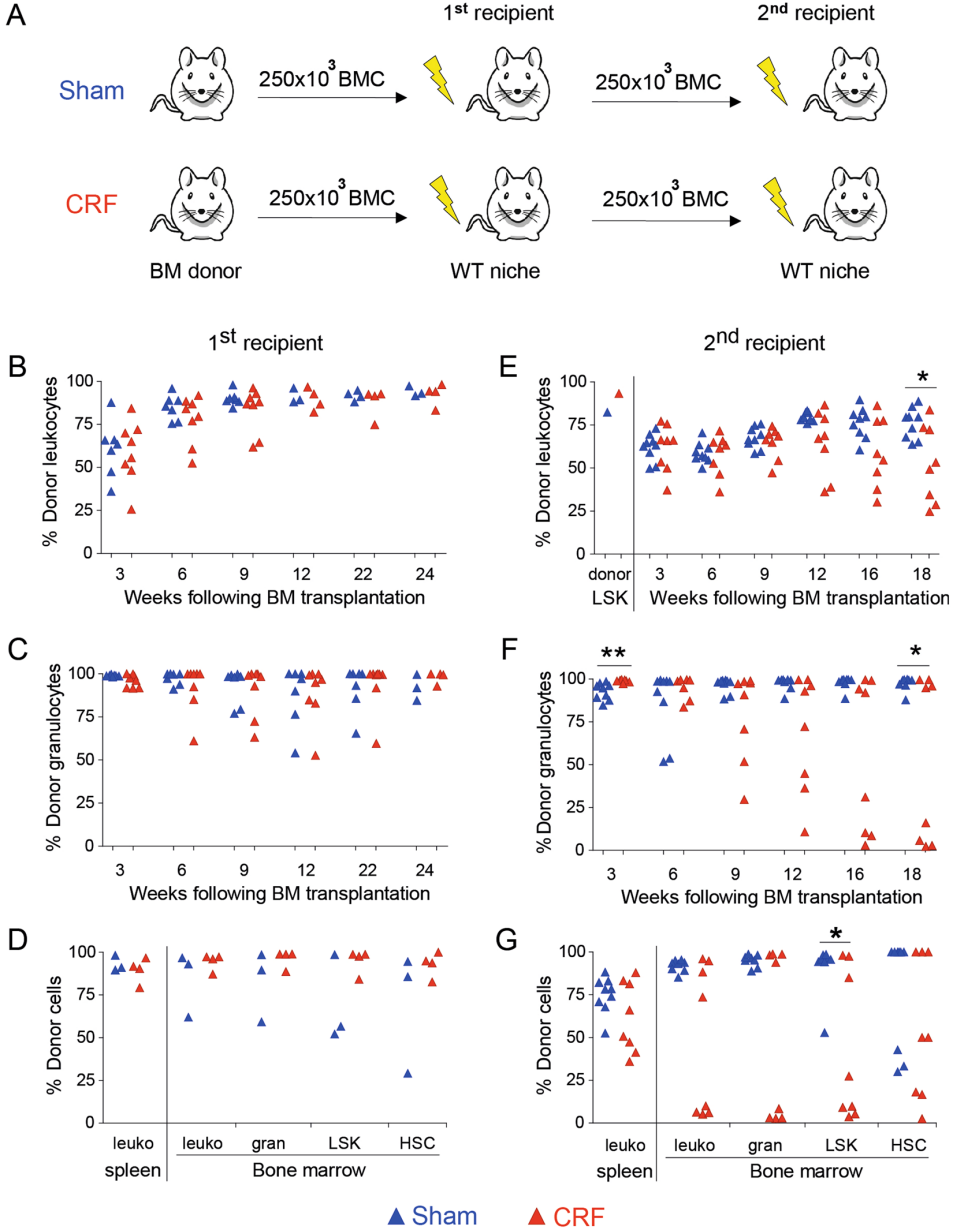
HSC compartment is impaired in CKD-MBD mice. (**A**) BMC obtained from CKD-MBD mice and sham-operated controls were harvested and transplanted into healthy, lethally irradiated, congenic recipients. At 24 weeks after transplantation, BMC obtained from primary recipients were harvested, pooled, analyzed for LSK chimerism (**E**), and re-transplanted into lethally irradiated secondary recipients. At three-week intervals, donor chimerism was analyzed in (**B–D**) primary and (**E–G**) secondary recipients. (**B**,**E**) Donor-derived CD45^+^ cells and (**C**,**F**) granulocytes were assessed. (**D**,**G**) At 18–24 weeks after transplantation the percentage of donor-derived CD45^+^ cells, granulocytes, Lin^neg^Sca-1^pos^c-kit^hi^ (LSK) cells and CD34^neg^LSK (HSC) in the spleen and BM were analyzed. ^*^p < 0.05 and ^**^p < 0.01. In panel B–G, each dot represents a single mouse.

To investigate whether the decrease in HSC frequencies in CRF mice *in vitro* also points towards a functional defect *in vivo*, transplantation studies were performed. BM cells obtained from CRF donors and sham-operated controls were harvested at week 12 following nephrectomy and 250 × 10^3^ BM cells were transplanted into lethally irradiated, congenic recipients (Fig. 3A). Chimerism levels in PB, spleen and BM were similar for recipients of CRF or control BM (Fig. 3B–D). To assess whether BM obtained from CRF donors exhibited long-term repopulation potential, secondary transplantations were performed. Four primary recipients of CRF BM and three primary recipients of control BM were selected as donors. The CRF donors had slightly higher levels of donor cells in the PB compared to sham-operated controls (CRF: 94% CD45^+^ chimerism; controls: 84% CD45^+^ chimerism). Pooled BM cells obtained from each donor group were retransplanted into lethally irradiated, secondary recipients. From week 9 after secondary transplantation onwards, both CD45^+^ and granulocyte chimerism was decreased in CRF secondary recipients compared to secondary recipients of control BM (Fig. 3E,F). Moreover, at 18 weeks following retransplantation, donor granulocyte frequencies decreased to 5.5% ± 6.4% donor cells in 50% of the secondary recipients of CRF BM. In contrast, all secondary recipients of control BM were highly chimeric for donor granulocytes (Fig. 3F). Furthermore, at 18 weeks following secondary transplant, decreased levels of donor LSK and LSK-CD34^−^ BM cells were found in secondary recipients of CRF BM compared to controls (Fig. 3G). Together, these *in vivo* data indicate that the HSC compartment of CRF mice has impaired repopulating potential.

### The HSC microenvironment is functionally impaired in CKD-MBD

**Figure 4:**
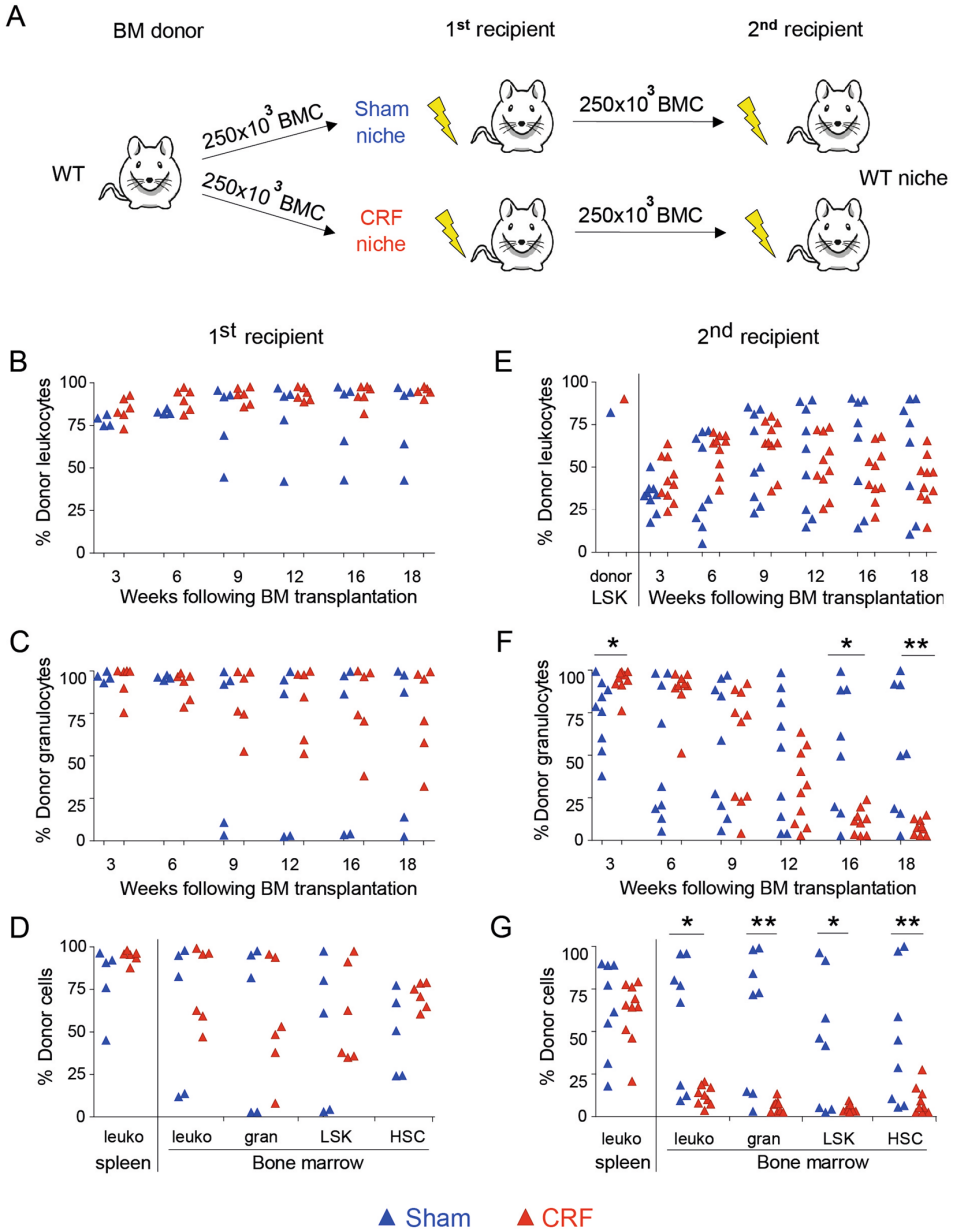
The HSC niche of CKD-MBD mice is functionally impaired and affects the repopulating capacity of HSC. (**A**) BM cells obtained from untreated donors were harvested and transplanted into lethally irradiated, congenic CKD-MBD or sham-operated recipients. At 18 weeks after transplantation, BMC obtained from primary recipients were harvested, pooled, analyzed for LSK chimerism (**E**) and re-transplanted into lethally irradiated secondary recipients. At three-week intervals, donor chimerism was analyzed in (**B–D**) primary and (**E–G**) secondary recipients. Donor-derived (**B**,**E**) CD45^+^ cells and (**C**,**F**) granulocytes were assessed. (**D**,**G**) At 18 weeks after transplantation the percentage of donor-derived CD45^+^ cells, granulocytes, Lin^neg^Sca-1^pos^c-kit^hi^ (LSK) cells and CD34^neg^LSK (HSC) in the spleen and BM were analyzed. ^*^p < 0.05 and ^**^p < 0.01. In panel B–G, each dot represents a single mouse.

To study whether CRF directly affects the repopulating capacity of HSC or indirectly impacts HSC through the microenvironment, BM cells obtained from healthy donors were transplanted into lethally irradiated CRF recipients or sham-operated controls (Fig. 4A). At 18 weeks following transplantation similar donor chimerism was observed for CRF and sham-operated recipients (Fig. 4B–D). To assess whether the CRF microenvironment affected the long-term repopulating capacity of HSC, secondary transplantations were performed. Of each recipient group (CRF or control), three primary recipients with the highest donor chimerism levels were selected as BM cell donors for secondary transplantation. The selected donors had similar levels of donor cells in the PB (CRF primary recipients: 97% CD45^+^ chimerism; sham-operated primary recipients: 91% CD45^+^ chimerism). Pooled donor BM cells were retransplanted into healthy, lethally irradiated, secondary recipients (Fig. 4A). From 9 weeks after transplantation onwards, donor granulocyte chimerism decreased in the secondary recipients of CRF BM (Fig. 4F). At 18 weeks following retransplantation, the PB of secondary recipients of CRF BM was largely depleted for donor granulocytes. Moreover, the BM of secondary recipients of CRF donor BM was almost completely devoid of donor cells (Fig. 4G). This is in sharp contrast with recipients of BM obtained from sham-operated control donors as most of these recipients had still donor cells in their PB, spleen and BM (Fig. 4E–G).

Together, these data indicate that the loss of long-term repopulating capacity of CRF BM is associated with a functionally impaired HSC niche.

### Quiescent HSC are decreased in CKD-MBD

**Figure 5:**
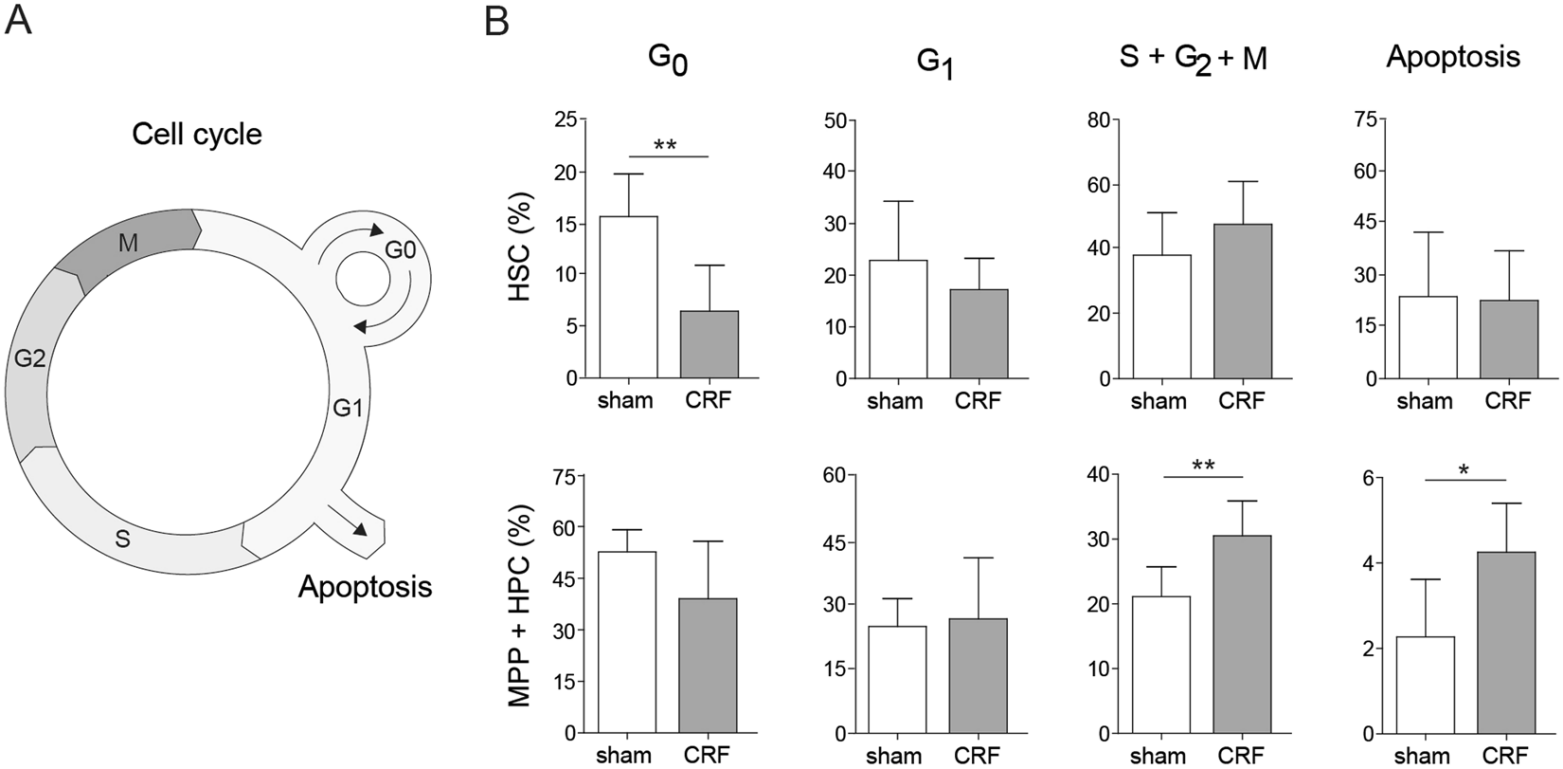
Quiescent HSC are decreased in CRF mice. At 12 weeks after nephrectomy, BM cells obtained from CRF and sham-operated controls were harvested and analyzed for the cell cycle status. (**A**) Schematic overview of the cell cycle. (**B**) Frequency of MPP/HPC and HSC in different stages of cell cycle. N = 7–8 per group. Means ± SD are depicted. ^*^p < 0.05 ^**^p < 0.01.

To investigate whether a change in the cell cycle state of HSC explains the hematopoietic exhaustion that we observe following transplantation of BM cells obtained from CRF donors, BM was analyzed for cell cycle state (Fig. 5A). In CRF mice the frequency of quiescent G0 HSC was 50% decreased, coinciding with a significant increase HPC and MPP that are in the S/G2/M-phase in CRF mice (Fig. 5B). Moreover, apoptotic HPC and MPP were significantly increased in CRF mice. Given these observations, we propose that the increase in non-quiescent HSC in CRF may explain the depletion of HSC following sequential transplantations.

### CKD-MBD impairs osteal macrophages and B-lymphopoiesis

**Figure 6:**
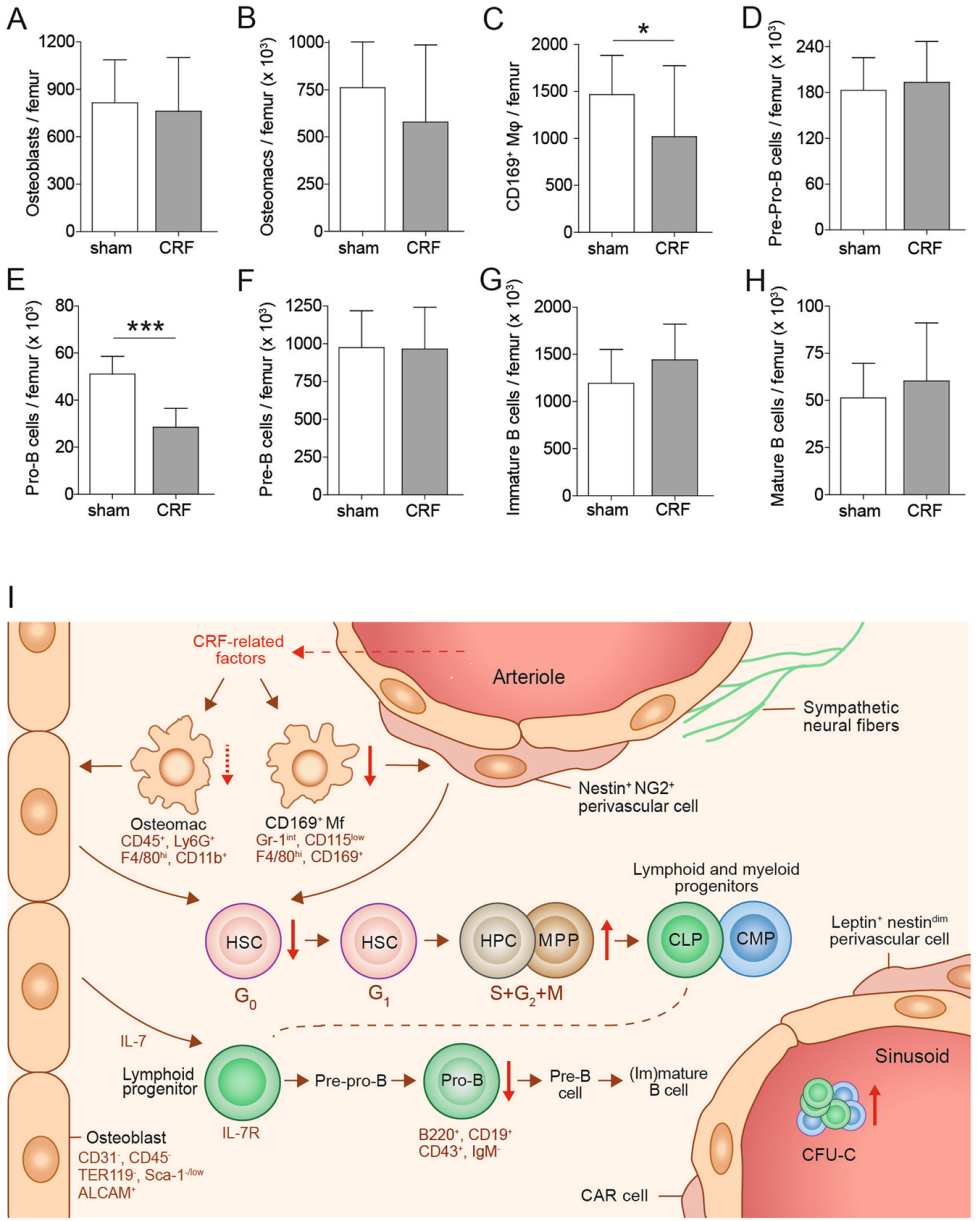
CKD-MBD impairs osteal macrophages and B-lymphopoiesis. (**A**) absolute number of CD31^−^CD45^−^TER119^−^Sca-1^−/lo^ALCAM^+^ osteoblasts was determined in collagenase-treated bones. (**B**) The absolute number of CD45^+^Ly6G^+^F4/80^+^CD11b^+^ osteal macrophages (osteomacs) and (**C**) CD169^+^Gr-1^int^CD115^low^F4/80^+^ macrophages (CD169^+^ Mɸ) were determined in CKD-MBD and sham-operated control BM cells. (**D–H**) The absolute number of B cells at each maturation stage was enumerated. (I) A schematic overview of the HSC niche after induction of CKD-MBD. For all experiments: n = 7–8 per group ^*^0.01 < p < 0.05, ^***^p < 0.0001. In A-D, the total cell number per femur is depicted, based on the percentages obtained by flow cytometric analysis and the total cell count per femur of each individual mouse.

To investigate the contribution of osteoblasts to the exhaustion of HSC following sequential transplantation, we have enumerated the CD31^−^CD45^−^TER119^−^Sca-1^−/lo^ALCAM^+^ osteoblasts. As shown in Fig. 6A, the frequency of osteoblasts per femur remained unaltered. Osteal macrophages and CD169^+^ macrophages promote HSC retention in the BM^29,30^. To evaluate the role of these macrophages in HSC maintenance, we have enumerated the number of CD45^+^Ly6G^+^F4/80^+^CD11b^+^ osteal macrophages and of CD169^+^Gr-1^int^CD115^lo^F4/80^+^ macrophages. At 12 weeks following nephrectomy, the absolute numbers of osteal macrophages and of CD169^+^ macrophages were decreased in CRF mice compared to controls (Fig. 6B,C). Since these macrophages participate in the maintenance of osteoblasts and their precursors, a decrease in these populations may point towards a downstream defect in osteoblasts. As B-lymphopoiesis depends fully on osteoblasts^31^, differentiation stages of B-lymphopoiesis were analyzed by flow cytometry. Although pre-pro and pre-B cell numbers did not change in CRF compared to sham-operated controls (Fig. 6D,F–H), the absolute number of pro-B cells was significantly decreased in CRF compared to controls (Fig. 6E).

## Discussion

The tightly balanced HSC niche plays a key role in the maintenance of HSC. Endothelial cells and cells of the osteoblastic lineage have been identified as crucial components of this microenvironment^12,22–24,32,33^. We hypothesized that in CKD-MBD, endothelial injury and disturbed bone metabolism affect the HSC niche. This, in turn, may disturb stem- and progenitor cell homeostasis.

Thus far, the effects of CKD-MBD on the BM microenvironment have been underestimated. In our model for non-progressive CKD-MBD, we observed not only a significant decrease in EPC frequencies and increased osteoblast and osteoclast activity, but also a decrease in functional HSC. Upon transplantation into primary recipients, BM obtained from CRF donors exhibited multilineage repopulation capacity. However, the HSC in the transplant were impaired in their long-term repopulation potential as the repopulation capacity was lost at 9 weeks following secondary transplantation. This suggests a specific defect in the HSC compartment.

Increased levels of CFU-C were observed in the PB and spleen of CRF mice, pointing towards pathophysiologic alterations in the HSC microenvironment^28^. This observation was further strengthened by the finding that HSC had lost their long-term repopulating capacity during a temporary exposure to a CKD-MBD microenvironment. In contrast, HSC that were previously transplanted into sham-operated ‘healthy’ primary hosts were capable of long-term repopulation of secondary recipients. Together this points towards a severe impairment of the hematopoietic system in CRF.

The endosteal region of the BM contains two populations of macrophages that contribute to the HSC niche. Osteal macrophages support osteoblast differentiation and mineralization *in vitro* and contribute to the maintenance of HSC *in vivo* ^34^ and CD169^+^ macrophages promote retention of HSC and HPC in the niche^29^. Depletion of BM macrophages results in suppressed osteoblast function and HSC mobilization^29,30^. These macrophage populations were reduced in CRF mice. It remains however unclear, whether this depletion of BM macrophages is due to a direct effect of CRF on the macrophages or whether this is the result of osteoblast(precursor) changes in the CKD-MBD environment that in turn induce a reduction in macrophage numbers. We hypothesize that the decrease in niche-maintaining macrophages leads to a defect in osteoblast(precursor) function and impairment of the HSC niche. These niche defects lead to HSC mobilization towards the PB and spleen (Fig. 6I). However, the impact of the chronic systemic inflammatory state in CKD-MBD on the function of resident macrophages may provide an alternative mechanism that impairs HSC homeostasis.

CKD is characterized by a sustained release of PTH. A number of studies have implicated a role for PTH in the HSC niche. Administration of PTH increases the number of osteoblasts and recruits osteal macrophages to the site of bone remodeling^35^. Furthermore, PTH administration expands HPC without affecting HSC numbers^25^. It has also been shown that osteocytes respond to PTH. Changes in osteocytic activation result in decreased support of HSC^36^. Although this is in contrast with previously published data showing that PTH administration increases hemopoietic stem and progenitor cell numbers through PTH-receptor signalling in osteoblasts^25^, it indicates that PTH may act on different cell types with differential outcomes. It is therefore highly unlikely that osteoblasts are the sole population of osteolineage cells that are impaired in CRF. Long-term administration of Cinacalcet in our model decreased PTH levels, but did not increase HSC numbers. We therefore hypothesize that other mechanisms than increased PTH levels lead to niche changes in CKD-MBD.

The generation of new B lymphocytes, B lymphopoiesis, relies heavily on support from osteoblasts^31^. Defects in osteoblast metabolism are therefore not only reflected in bone turnover, but also in B lymphopoiesis. Our observation that the absolute number of pro-B cells are significantly decreased in CRF directly points towards a defect in osteoblasts. Since RANKL is known to support B-cell development our observation that RANKL levels are elevated while pro-B cell numbers decrease appears counter intuitive. However, during B lymphopoiesis, B cells go through several maturation stages that are distinguished by different receptors and adhesion molecules. As such B cell require not only RANKL, but also interleukin 7, which is produced by osteoblasts. Changes is osteoblast metabolism may thus not only affect RANKL, but also the secretion of other proteins and cytokines required for B cell differentiation.

In addition, we observed an increase in CD31^−^CD45^−^Ter119^−^Sca-1^+^CD29^+^Nestin^+^ cells in CRF (MA and MvP, data not shown), a population that has previously been shown to expand in response to PTH^14^ and are located in the vicinity of CD169^+^ macrophages^29^. Together, this suggests that other (osteolineage) cells may also play a role in the observed HSC defect in our model.

The CRF-induced defect in the HSC niche results in a reduced capacity to maintain HSC in a quiescent state. As soon as HSC exit quiescence and enter G1, their capacity to functionally engraft irradiated recipients dramatically decreases^9^. Thus, the observed increase in cycling HSC in CKD-MBD may also contribute to the observed loss in repopulating potential of HSC obtained from CKD-MBD microenvironments.

Long-term dysfunction of the HSC niche may also be a causal factor in the endothelial dysfunction that is invariably related to CKD and its associated elevated risk for cardiovascular disease^3^. In addition, impaired HSC maintenance provides a plausible explanation for the decreased number of CD34^+^ HSPC in the PB of CKD patients^37^. Evidence is accumulating that multiple BM-derived cell populations including CD34^+^ HSPC can promote endothelial repair and regeneration in conditions of pro-atherogenic macrovascular endothelial cell injury^6^ as well as in microvascular injury associated with ischemia reperfusion injury^5^. Impairment of HSC maintenance due to CRF induced bone defects may lead to a depletion of this CD34^+^ population with regenerative potential.

## Materials and Methods

### Animals

Eight to 12-week-old female C57BL/6-Ly5.2 and C57BL/6-Ly5.1 mice were obtained from Charles River Laboratories (Maastricht, The Netherlands). The animals were fed commercial rodent chow and acidified water ad libitum and were maintained in the animal facility of the Leiden University Medical Center under conventional conditions. In selected experiments, Cinacalcet was administered as an additive in rodent chow to CKD and sham controls from week 6 after CKD induction onwards. All mouse studies were approved and performed according to the guidelines of the relevant authorities of the Leiden University Medical Center/Leiden University the Netherlands and within the guidelines set by the Dutch government.

### Induction of chronic kidney disease

To induce CKD, a 2-step surgical procedure was performed as previously described^26^. Briefly, through a 1.5 cm flank incision the right kidney was decapsulated and its cortex was cauterized. Two weeks later, the left kidney was decapsulated and nephrectomized. During both surgeries, damage of adrenal glands was avoided. Control mice underwent sham operations (anesthesia, incision and taking the kidney out of the abdomen) at the same time points. Sham group underwent the same surgical procedure, but the clamp was not used.

### Blood plasma analysis

Blood was collected from the tail vein into the lithium-heparin precoated Microvette CB 300 tubes (Sarstedt, Germany). PB cells and hemoglobin levels were measured using a Sysmex F-820 analyzer (Sysmex, Etten-Leur, the Netherlands). PB plasma was stored at −20 °C until analysis. Plasma levels of total calcium, phosphorus, creatinine and urea were assessed using the P800 modular analyzer (Roche diagnostics, Indianapolis, USA). Intact PTH was quantified using the quantitative ELISA kit (Immunotopics, San Clemente, USA) according to the manufacturer’s recommendations.

### Micro-CT scan

Bone changes in the metaphysis region were monitored using the Sky-Scan 1076 *ex vivo* x-ray microtomograph. All samples were *ex vivo* scanned and reconstructed according to the same settings using Skyscan software. Next, all scans were converted to binary (black-and-white) datasets using local thresholds^38,39^. Trabecular and cortical bone was separated using automated in-house software^40^. Using these datasets, we calculated BV/TV and SMI for trabecular bone. Trabecular bone volume fraction (BV/TV) was calculated as a 3-D morphometric parameter representing the ratio of trabecular bone volume (BV; in mm^3^) to endocortical tissue volume (TV; in mm^3^). The structure model index (SMI) is calculated based on a differential analysis of the triangulated surface of a structure and defined as six trabecular bone volumes (BV; in mm^3^) to squared bone surface area (BS; in mm^2^) multiply by dS/dr, where dS/dr is the surface area derivative with respect to a linear measure r, corresponding to the half thickness or the radius assumed constant over the entire structure.

### Antibodies for cell analysis

All antibodies used are described in Supplemental Table 1. Cells were analyzed on a Canto II with Diva software (BD Biosciences, Erebodegem, Belgium). To evaluate cell cycle status, BM cells were stained for lineage markers, Sca-1, c-Kit and CD34. Next, the cells were fixed and permeabilized (fix and perm kit, eBioscience, Vienna, Austria) and stained with anti-Ki67 and DAPI. Isotype-labeled BM cells served as controls to gate Ki67 negative cells. G0 cells show low DAPI staining and are Ki67^−^, G1 cells are DAPI^low^Ki67^+^, G2/S/M cells are DAPI^HI^Ki67^+^. Apoptotic cells are also identified in the same staining as DAPI^−^ cells.

### Preparation of cell suspensions

Using sterile procedures, BM and spleen cells were obtained as previously described^41^.

#### Progenitor cell assays

Colony Forming Units (CFU) were cultured as described previously^41^. After sacrificing the mice, we determined the number of peripheral blood cells per ml as well and the total cell number of the femurs and spleens of each individual mouse. To determine the Colony Forming Units, we cultured 5 × 10^5^ peripheral blood cells, 5 × 10^4^ bone marrow cells and 1 × 10^6^ spleen cells in semisolid medium containing GM-CSF, G-CSF, IL-3 and EPO. After 6 days of culture in a fully humidified atmosphere of 37 °C 5% CO2, the number of colonies (defined as an aggregate of ≥20 cells) were scored using an inverted light microscope. Next, the number of CFU-C was calculated using the number of CFU-C and the cell counts that were obtained in each tissue.

#### Cobblestone area forming cell (CAFC) assay

CAFC frequencies were determined as previously described^42^. To assay a particular cell suspension, we used 8 dilution steps differing with a factor of 2.5, with 15 wells per dilution. The cells were cultured at 33 °C, 7% CO_2_ and were fed weekly by changing half of the medium. All wells were inspected at weekly intervals and scored positive if at least one phase-dark hematopoietic clone (cobblestone area, at least 5 cells) was observed. The CAFC frequencies were calculated using Poisson statistics.

#### Bone marrow cell transplantation

Recipient mice were irradiated in perspex chambers using an Orthovolt (Xstrahl medical, Walsall, UK). All recipients received TBI at a dose 9.5 Gy (TBI; lethal irradiation) was given as a single dose. Four hours following TBI, BM cells were injected at indicated numbers via caudal vein injection in 0.2 ml of saline, containing 0.2% bovine serum albumin. At different time points after transplantation, peripheral blood samples were drawn from the tail vein to assess donor chimerism.

### Statistical analysis

All values are presented as mean with standard errors of the mean (SEM). Treated and untreated groups were compared using the unpaired t-test and non-parametric Mann-Whitney test, as appropriate for normal-distributed and skewed data, correspondingly. All statistical calculations were performed using GraphPad Prism software (La Jolla, California, USA). P ≤ 0.05 was considered statistically significant. All raw data is available on request.

## Electronic supplementary material

Supplementary info and data
