# Supplementary material for: Chronic kidney failure mineral bone disorder leads to a permanent loss of hematopoietic stem cells through dysfunction of the stem cell niche
Source: Sci Rep. 2018 Oct 18;8:15385. doi: 10.1038/s41598-018-33979-7 (PMC6194087; doi:10.1038/s41598-018-33979-7)

**Chronic kidney failure mineral bone disorder leads to a permanent loss of hematopoietic stem cells through dysfunction of the stem cell niche**

Marina A. Aleksinskaya^1*^, Matthieu Monge^1*^, Michiel Siebelt^2^, Edith M. Slot^3^, Karin Koekkoek^1^, Ruben G. de Bruin^1^, Ziad Massy^4^, Harrie Weinans^5,6^, Ton J. Rabelink^1^, Willem E. Fibbe^3^, Anton Jan van Zonneveld ^1ǂ^ and Melissa van *Pel^3ǂ^*

**Supplementary methods and data sets**

**Supplementary methods to Aleksinskaya et al.**

After obtaining the bone marrow cells by flushing femurs with RPMI supplemented with 2% FCS, penicillin, streptomycin and L-glutamine, the bone marrow cells were lysed using Trizol reagent (Invitrogen). RNA was obtained using the RNeasy mini kit (Qiagen) according to the manufacturers recommendations and subsequently cDNA was generated using oligo(dT) primers (Invitrogen). The following primers were used: RANKL: fw-CACCATCAGCTGAAGATAGT, rev-CCAAGATCTCTAACATGACG; M-CSF: fw-agtattgccaaggaggtgtcag, rev-atcttggcatgaagtctccattt; CatK: fw-TCTGCTGCACGTATTGGAAG, rev-GGCCTCTCTTGGTGTCCATA. Beta-Actin: fw: AGACCTCTATGCCAACACAG, rev: TAGGAGCCAGAGCAGTAATC. Quantative Real Time-Polymerase Chain Reaction (qRT-PCR) was performed using SYBR Green mastermix (Bio-Rad Laboratories, Veenendaal, The Netherlands). Relative gene expression was calculated using the comparative threshold cycle (C_T_) method, with *β-actin* as the endogenous reference gene.


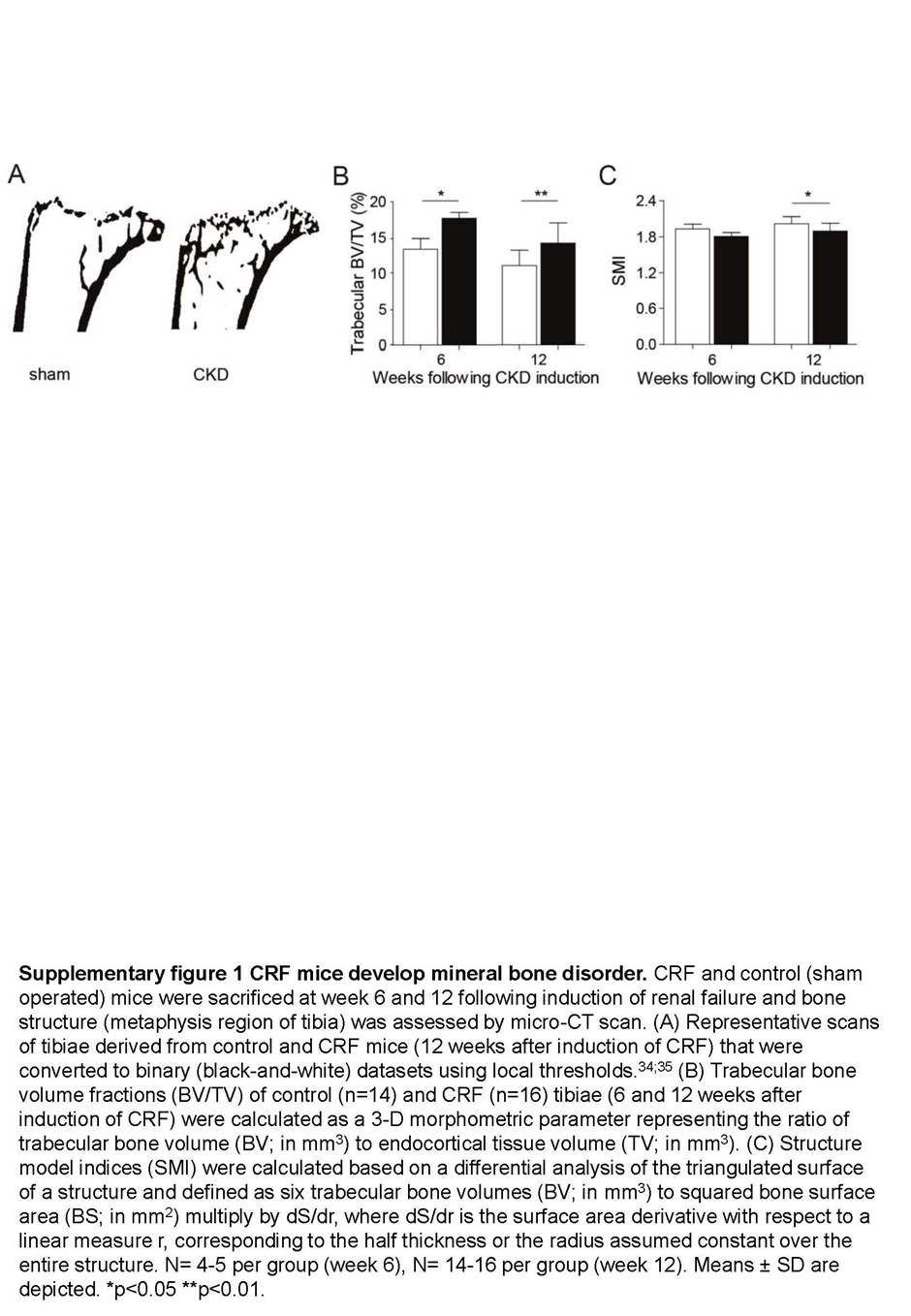


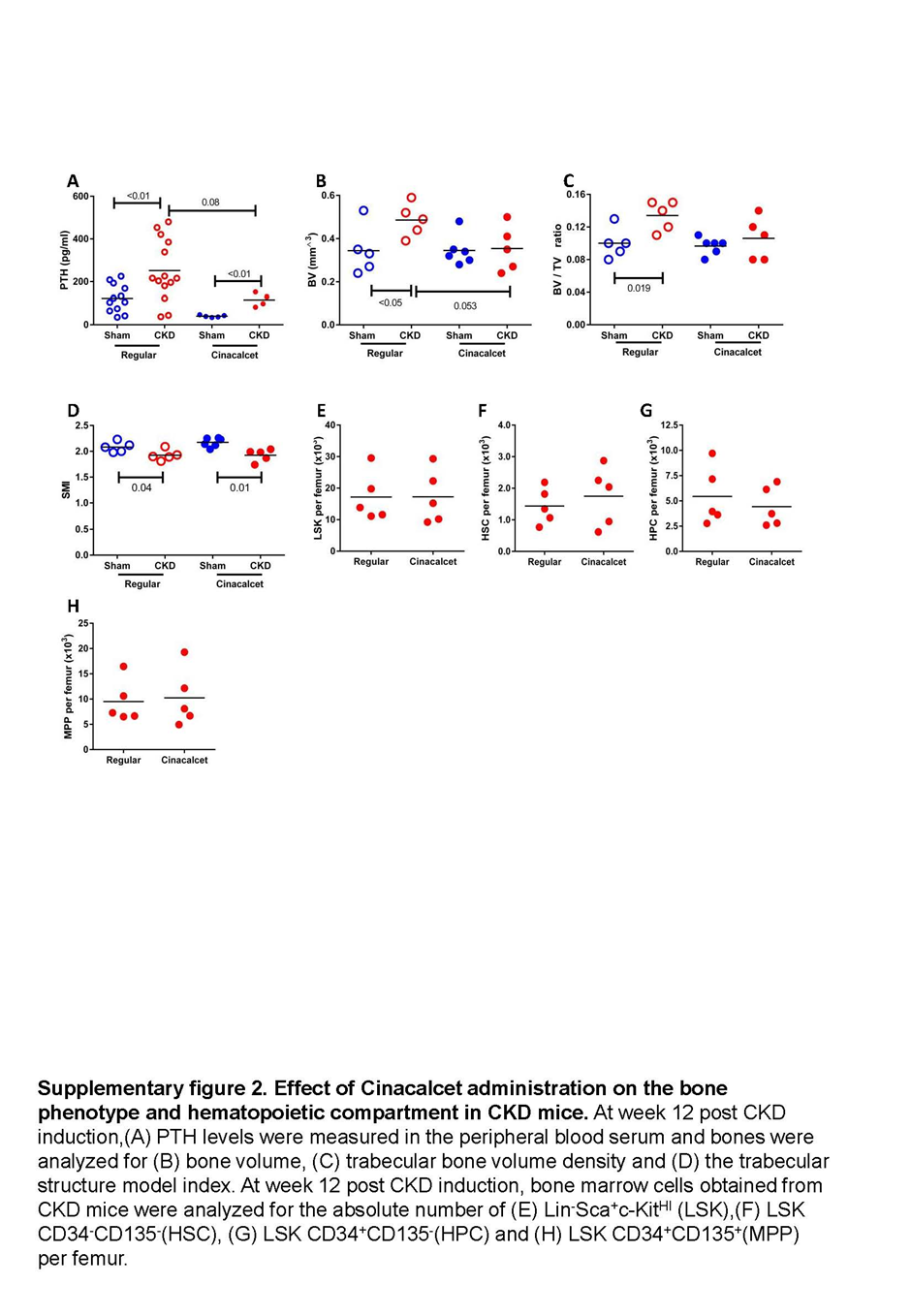


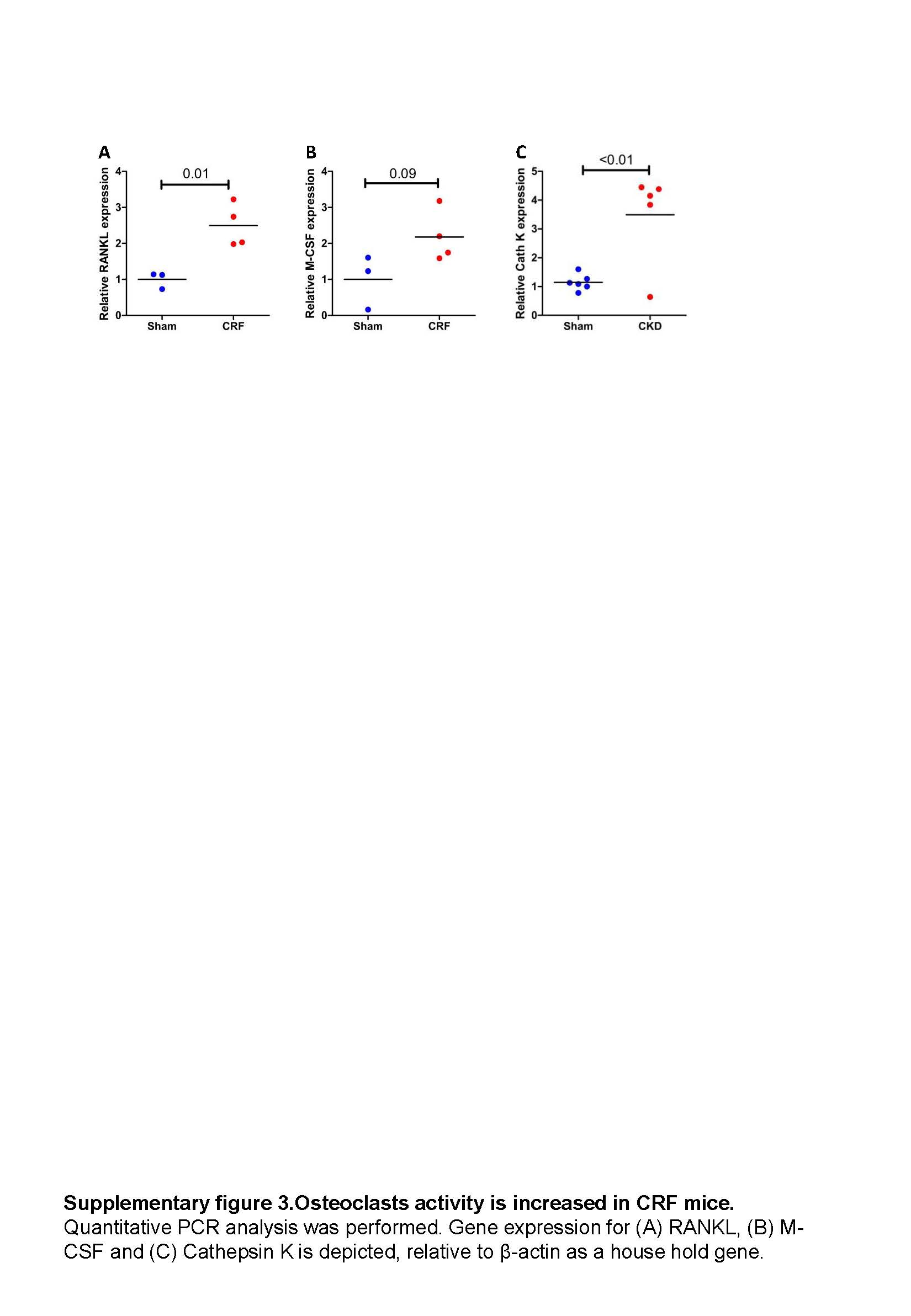

Supplement: Supplementary file 1 — Supplementary info and data [file 41598_2018_33979_MOESM1_ESM.doc]
